# Enhancing Imaging Anatomy Competency: Integrating Digital Imaging and Communications in Medicine (DICOM) Viewers Into the Anatomy Lab Experience

**DOI:** 10.7759/cureus.68878

**Published:** 2024-09-07

**Authors:** Luke Worley, Maria A Colley, Caroline C Rodriguez, David Redden, Drew Logullo, William Pearson

**Affiliations:** 1 Anatomical Sciences, Edward Via College of Osteopathic Medicine, Auburn, USA; 2 Research and Biostatistics, Edward Via College of Osteopathic Medicine, Auburn, USA; 3 Biomedical Affairs and Research, Edward Via College of Osteopathic Medicine, Auburn, USA

**Keywords:** anatomical education, assessing competencies, eye-tracking, imaging anatomy, medical education

## Abstract

Introduction: Radiologic interpretation is a skill necessary for all physicians to provide quality care for their patients. However, some medical students are not exposed to Digital Imaging and Communications in Medicine (DICOM) imaging manipulation until their third year during clinical rotations. The objective of this study is to evaluate how medical students exposed to DICOM manipulation perform on identifying anatomical structures compared to students who were not exposed.

Methods: This was a cross-sectional cohort study with 19 medical student participants organized into a test and control group. The test group consisted of first-year students who had been exposed to a new imaging anatomy curriculum (n = 9). The control group consisted of second-year students who had not had this experience (n = 10). The outcomes measured included quiz performance, self-reported confidence levels, and eye-tracking data.

Results: Students in the test group performed better on the quiz compared to students in the control group (p = 0.03). Confidence between the test and control groups was not significantly different (p = 0.16), though a moderate to large effect size difference was noted (Hedges’ g = 0.75). Saccade peak velocity and fixation duration between the groups were not significantly different (p = 0.29, p = 0.77), though a moderate effect size improvement was noted in saccade peak velocity for the test group (Hedges’ g = 0.49).

Conclusion: The results from this study suggest that the early introduction of DICOM imaging into a medical school curriculum does impact students’ performance when asked to identify anatomical structures on a standardized quiz.

## Introduction

With the introduction of the integrated model curriculum across the US, approaches to learning why and how the basic sciences are incorporated into clinical decisions are constantly evolving to match the evolution of medical practice [1,2]. Preclinical curriculums have varied approaches for integrating radiological interpretation into preclinical courses and clinical clerkships [3]. Often, radiology is utilized as adjunct material for anatomy and/or pathology courses and mostly as static images [1]. Within preclinical education, there is currently a lack of exposure to practice using Digital Imaging and Communications in Medicine (DICOM), the international standard for medical images. Students are also limited to passive exposure to dynamic images in their clinical clerkships. Studies have shown that medical students introduced to radiology earlier in the curriculum have improved their perception of the importance of medical imaging, as well as increased interest in the specialty as a whole [4-6]. Another study, which surveyed first-year residents (PGY-1), found that approximately two-thirds of interns were expected to make preliminary interpretations of imaging studies in their role [7]. These findings support the importance of developing learner competence in using and interpreting DICOM imaging data within the medical school curriculum.

Diagnostic imaging is an important tool for the practicing physician regardless of their respective specialty [8, 9]. A survey of 331 clinicians indicated medical imaging is highly valued and estimated that usage will further increase in the next 10 years. Furthermore, this survey revealed that 40.6% (134) of clinicians interpret a majority of their imaging studies without consulting a radiologist or reading the associated report [10]. The increased availability of imaging allows for more efficient patient care and the ability for physicians to read their own ordered studies, while also reducing the cost burden to patients. This exemplifies why it is important for medical students to become competent with imaging data interaction. Failure to reach this competency level during training may lead to overutilization of healthcare resources [11-13].

Developing competency is an important aspect of the medical school curriculum that is mainly achieved by performance benchmarking and feedback [14-16]. Without the appropriate learning opportunity, students may struggle to gain the confidence and abilities to grow into clinicians. Research has shown that spaced practice is superior to massed practice in knowledge acquisition and long-term retention [16, 17]. However, many exams rely on recognition (multiple-choice questions) rather than retrieval (short answer) [17]. This may lead to students focusing on testing performance rather than developing habits, such as recognizing morphology in medical imaging, necessary to become competent physicians.

Systematic interpretation of medical images is a complex skill that requires anatomical knowledge, visual pattern recognition, pathological process knowledge, and patient-specific information [18-20]. Because imaging in the medical school curriculum is often limited to static images, and because in-house and board exams generally utilize multiple-choice formats, it is necessary to evaluate whether students are learning to systematically interpret images rather than relying on memorization of radiographic findings. Without objective data to observe student performance, it is almost impossible to know how students are reading images presented on exams or during clinical clerkships. The introduction of eye-tracking software has allowed for the study of conscious and subconscious human behavior by analyzing gaze patterns and visual-spatial evaluations [19]. Eye-tracking has already been used to study the performance of physicians in a variety of ways, such as studies demonstrating the differences between gaze patterns in experts and novices [21-23]. It has also been postulated that eye-tracking can be used within medical education as a feedback mechanism for faculty [19]. This technology allows the opportunity to objectively analyze how an individual interacts with imaging anatomy in various modalities.

The goal of this pilot study was to gather preliminary data to test the impact of an imaging anatomy curriculum where learners practice identifying anatomy using DICOM viewers versus preparing for assessments by memorizing static images. Further, it aims to uncover whether this new approach improves imaging knowledge acquisition and if students are improving upon pre-formed skills. To reach this goal, we compared the outcomes of students exposed to DICOM manipulation with those of students who were not exposed by the following means: 1) quiz performance, 2) self-reported level of confidence, and 3) eye-tracking data.

## Materials and methods

Ethics approval for this study was granted by the Edward Via College of Osteopathic Medicine (VCOM) Institutional Review Board, Blacksburg, VA (IRB # 2022-086). Informed consent was obtained prior to the start of each study session. No compensation was provided to participants.

Nineteen subjects were recruited among first-year or second-year medical students. VCOM Auburn has a one-pass integrated system over eight blocks in two academic years. A gross anatomy course is integrated into the first five of those blocks, including 1) osteology, 2) musculoskeletal, 3) head and neck, 4) cardiothoracic, and 5) abdominopelvic. Second-year medical students were assigned to the control group (n = 10). The control group memorized labeled PowerPoint images (Microsoft Corp., Redmond, WA) in preparation for practical lab exams. First-year medical students were assigned to the test group (n = 9) as their cohort was exposed to a new DICOM imaging manipulation curriculum from the beginning of their anatomy course. The test group’s anatomy lab experience included using a Diagnostic DICOM viewer (PostDICOM, Herten, The Netherlands) to identify structures in imaging studies relevant to the day's lab dissection. Curated, de-identified datasets of various modalities representing normal anatomy were provided to students on Canvas and lab computers. Learners attended a workshop led by second-year anatomy interns to help them download a DICOM viewer to a personal computer and learn how to use the software to identify images. During lab time, students worked on identifying structures on an imaging checklist that was included along with a checklist of structures to be identified on donor bodies. As a formative assessment, teams submitted files of labeled structures for a grade. Radiology reviews, held by faculty or anatomy interns, were offered to facilitate learning to navigate imaging using a DICOM viewer. Images used in the practical laboratory exam were taken from these datasets to incentivize student use. These multiple efforts were offered to provide opportunities for spaced-time retrieval of information as well as effortful learning. 

The amount of time elapsed from exposure to the imaging curriculum to data collection for the control group was nine months, whereas, for the test group, it was a range of two to five months with a mean equal to three months. A pre-quiz survey was administered to assess student perceptions of confidence in identifying anatomical structures in cardiothoracic imaging using a 10-point Likert scale (1 = not confident at all; 10 = very confident). We asked an open-ended question about the amount of time invested in learning imaging anatomy; however, the imprecision of this question undermined including these data for comparison. Participants were then asked to identify 15 anatomical structures on digital radiographic images and type their answers in a box. During the assessment, participant eye-tracking data were collected by an external device called AI-X (Smart Eye, Gothenburg, Sweden), and fixation duration and saccade peak velocity data were calculated using iMotions software (Copenhagen, Denmark).

To evaluate group differences in quiz scores and confidence, the Wilcoxon rank-sum test was used. Saccade peak velocity and fixation duration were measured multiple times per participant across all 15 images; therefore, generalized estimating equations were used to test for differences between the two groups. Effect sizes were reported as Hedges’ g.

## Results

**Figure 1:**
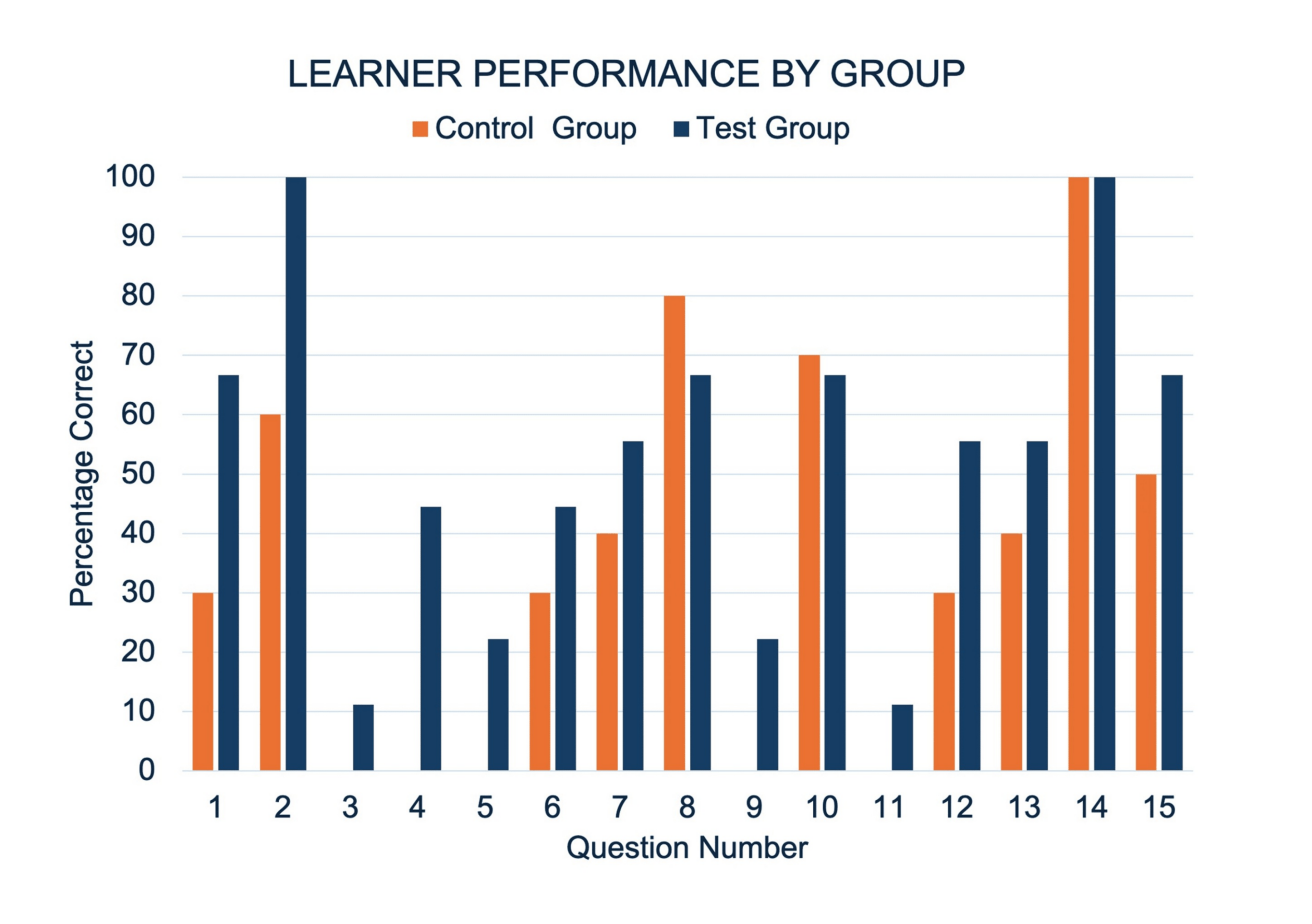
Percentage of Correct Responses for Each Group by Quiz Item Radiograph Quiz Items and Associated Structures: 1 = Left Third Rib, 7 = Right Main Bronchus, 14 = Right Hemidiaphragm, 15 = Aortic Knob Contract Enhanced Ct Quiz Items and Associated Structures: 2 = Esophagus, 3 = Oblique Fissure of the Right Lung, 4 = Left Superior Lobar Bronchus, 5 = Circumflex Branch of the Left Coronary Artery, 6 = Left Atrium, 8 = Right Coronary Artery, 9 = Coronary Sinus, 10 = Aortic Semilunar Valve, 11 = Right Brachiocephalic Vein, 12 = Superior Vena Cava, 13 = Right Coronary Artery

**Figure 2:**
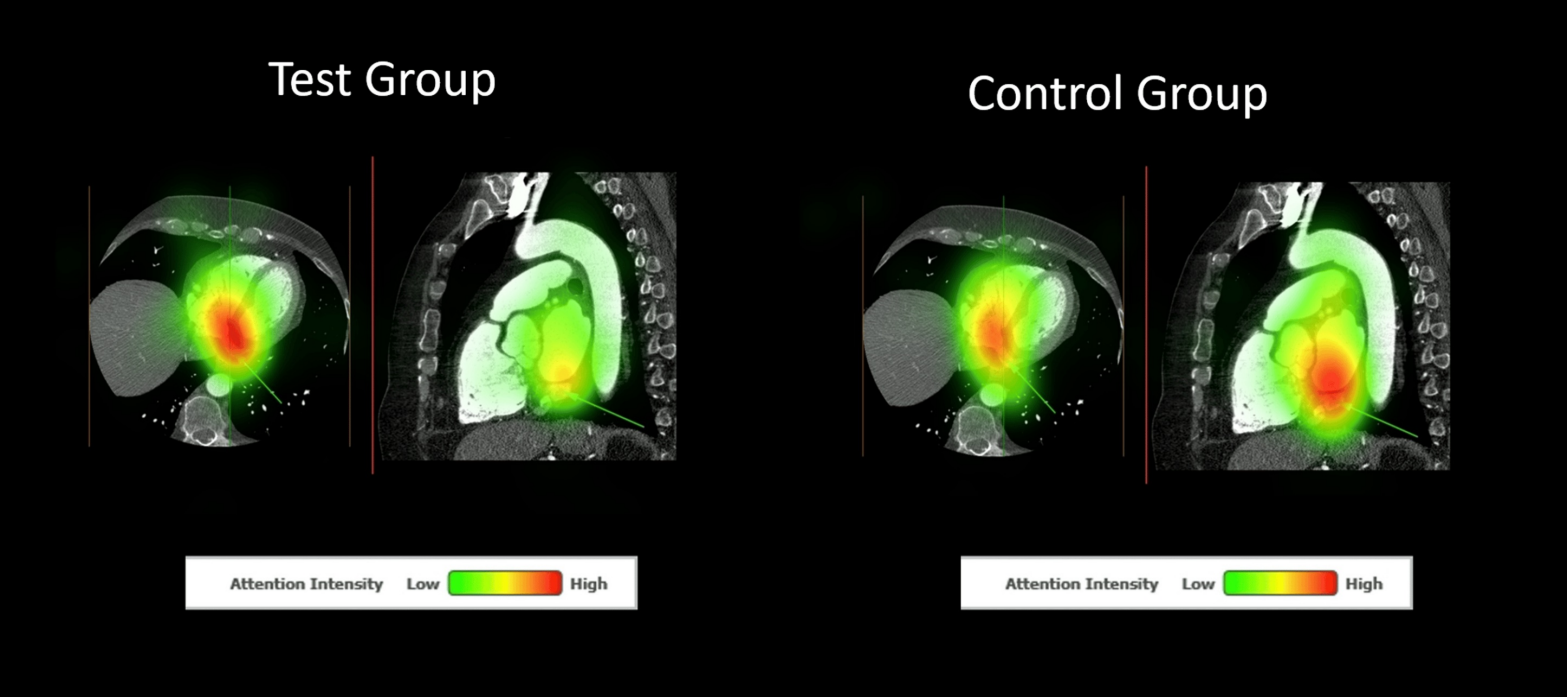
Heat Maps Depicting Combined Eye Fixation for Both Groups for Quiz Image 9 The Correct Answer for This Question Was “Coronary Sinus”.

**Figure 3:**
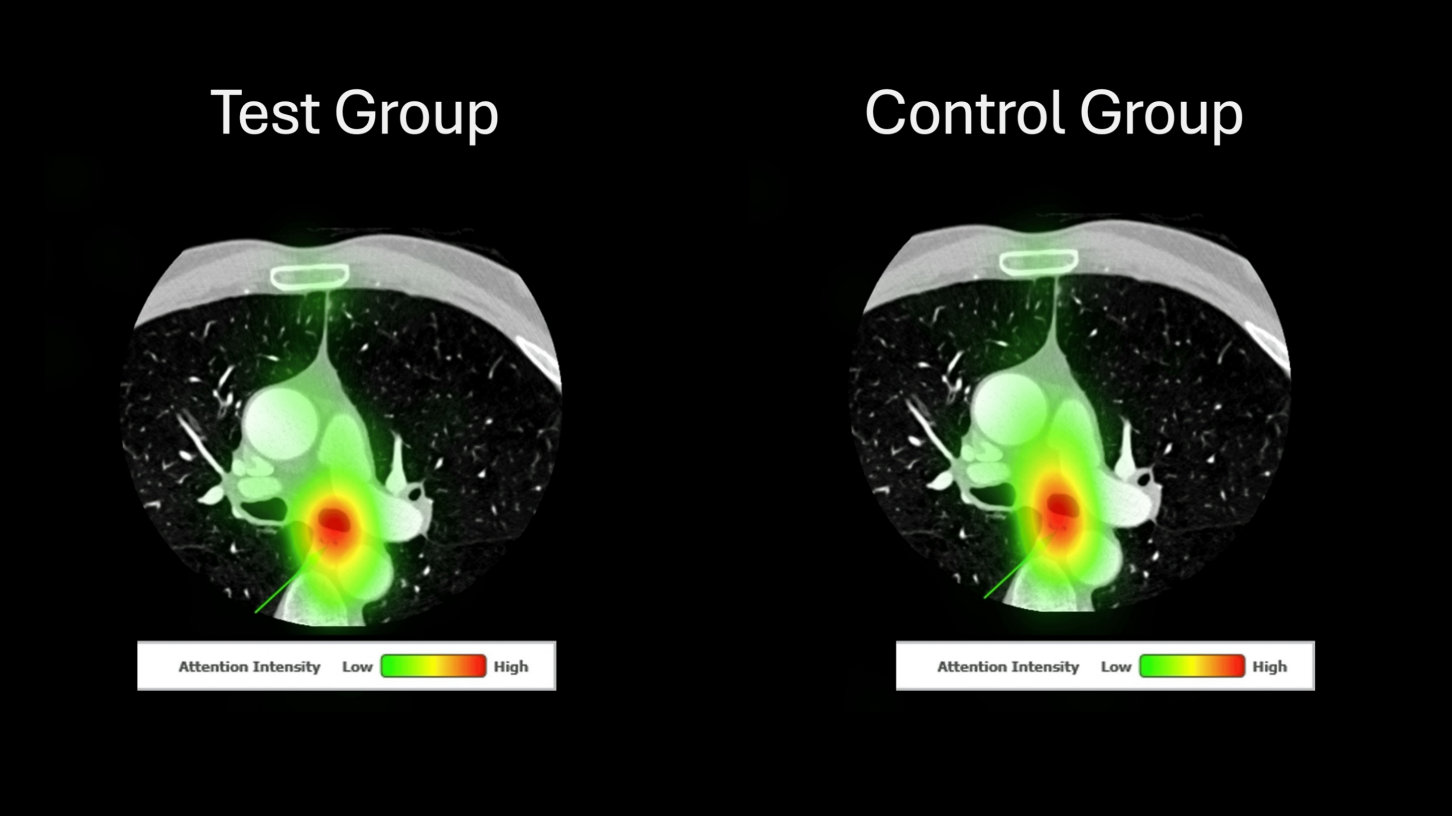
Heat Maps Depicting Combined Eye Fixation for Both Groups for Quiz Image 2 The Correct Answer for This Question Was “Esophagus”.

Table 1 presents outcome means for confidence, quiz score, fixation duration, and saccade peak velocity for both groups. Confidence between first-year (test group) and second-year (control group) learners was not significantly different. The test group performed better on the quiz than the control group, with a large effect size difference (Hedge’s g = 1.08). There was no statistically significant difference in saccade peak velocity and fixation duration between groups. Figure 1 showcases a comparison of the percentage correct for each question of the quiz between groups. Figures 2-3 present heat maps representing the mean eye-fixation duration for each group.

**Table 1:** Outcome Means and Standard Deviations by Group Significant Figures Are Indicated by an Asterisk.

	Test Group	Control Group	P-value	Hedges’ g
Quiz Performance	0.53 ± 0.18	0.37 ± 0.11	0.03*	1.08*
Perceived Confidence	7.00 ± 1.58	5.80 ± 1.62	0.16	0.75*
Fixation Duration	332.9 ±76.82	325.0 ± 52.23	0.77	0.12
Saccade Peak Velocity	134.7 ±20.10	126.2 ± 14.75	0.29	0.49*

## Discussion

This study found that participants in the test group using DICOM viewers to study imaging anatomy performed better than those who did not. However, in this small sample, there was no statistically significant difference in p-values of self-reported confidence levels or innate competency, as measured by eye tracking technology, despite some notable effect size differences.

Competency is achieved through deliberate practice, skill acquisition opportunities, adaptation to new information, and learning from past experiences. While a change in imaging anatomy curriculum is unlikely to produce expert-level competency, these data indicate improvements. This suggests that interactive opportunities using various imaging modalities can help learners develop unique skills in medical imaging and enhance their understanding of anatomy as they progress in their medical careers.

A limitation of this study was that the time lapse between educational exposure and data collection was not equivalent for the two groups. The additional time in between learning and testing in the control group may have introduced variability in their performance, confidence, and innate competency, as measured by eye tracking. While less time elapsed for the test group may have boosted their quiz performance, on the other hand, the control group had more experience in an integrated curriculum, which may have enhanced their quiz performance. Applying an Ebbinghaus forgetting curve equation predicts a 19.3% retention for the test group at a mean of three months and a 17.6% retention at nine months for the control group [24]. If the difference of 1.7% was added to the control group quiz mean, then the Hedges' g would be reduced from g = 1.08 to g =.97, which represents a large effect size difference in performance while accounting for forgetting.

A possible confounder in the present study is whether students practiced imaging skills using DICOM imaging or memorized images that others had labeled for exam preparation. It was reported that many learners relied on atlases created by a few peers rather than manipulating DICOM images themselves to prepare for the exam. Our innovations may only be as effective as the associated assessments among learners who, out of necessity, are experts in meeting expectations while conserving effort. To better train learners in imaging anatomy competency, the assessment paradigm needs to test learners' ability to identify structures using DICOM imaging. Following this study, a formative assessment was introduced that assesses the learner’s ability to demonstrate structures using DICOM imaging as part of a table conference [25].

A small sample size also limits this evaluation. While no statistically significant difference was found in student perception of confidence, a moderate to large effect size increase was found in favor of the test group (Hedges' g = 0.75). We also found a moderate effect size difference in saccade peak velocity (Hedges’ g = 0.49), indicating a change in innate imaging anatomy competency in the test group.

Using eye-tracking software to understand how medical learners analyze, interact with, and interpret medical images may offer valuable insights for medical educators about how to best train learners in imaging anatomy, though how to interpret eye-tracking findings is somewhat elusive [19]. We found a moderate effect size improvement in saccade peak velocity but not in fixation duration. Increased saccade velocity has been associated with increased arousal, which may indicate greater familiarity or engagement with images [26]. Fixation duration is thought to signal increased recognition, though other evidence indicates longer duration times may be associated with feature uncertainty [27, 28]. While using static images to assess eye tracking measures may mimic exam conditions, an arrow pointing to the structure to be identified likely confounds eye fixation duration measurement. Furthermore, these assessments do not simulate radiologic practice. A potential future application of eye tracking in assessing this innovation will involve capturing data as learners utilize a DICOM viewer to identify a specific structure.

## Conclusions

Preliminary data show that imaging anatomy curriculum where learners are provided DICOM images to manipulate for identifying anatomical structures has the potential to improve imaging anatomy competency. This finding demonstrates the value and importance of integrating interactive learning opportunities into the medical school curriculum. Future studies should consider assessing learners by having them identify structures by manipulating DICOM images rather than viewing static images, as it is more representative of their knowledge and expertise.
